# Role of protein intake in maintaining muscle mass composition among elderly females suffering from sarcopenia

**DOI:** 10.3389/fnut.2025.1547325

**Published:** 2025-05-12

**Authors:** Izwa Ishaq, Sana Noreen, Patrick Maduabuchi Aja, Ayomide Victor Atoki

**Affiliations:** ^1^University Institute of Diet and Nutritional Sciences, The University of Lahore, Lahore, Pakistan; ^2^Faculty of Biomedical Sciences, Kampala International University, Bushenyi, Uganda

**Keywords:** physical performance, magnetic resonance image, anthropometric measurements, sarcopenia, dietary protein

## Abstract

Dietary protein is crucial in preserving muscle mass and promoting long-term bone health, particularly in aging populations. The novelty of this study lies in evaluating the impact of varying protein intake levels (0.8 vs. 1.2 g/kg b.w/d) on muscle mass composition in elderly females suffering from sarcopenia. For this purpose, in this research trial, a total of 126 participants (60–75 years) were enrolled and equally divided into two groups: normal protein intake (0.8 g/kg b.w/d) and moderately high protein intake (1.2 g/kg b.w/d) for 12 weeks. The effects of dietary protein were assessed using anthropometric measurements, magnetic resonance imaging (MRI), handgrip, and knee flexion tests with baseline and post-intervention comparisons. Statistical analysis was conducted using SPSS, employing paired sample t-test at a significance level of *p* < 0.05. Results indicated a significant improvement in muscle mass composition with moderately high protein intake. Anthropometric parameters, including body mass (83.51 ± 4.23 kg) and waist circumference (113.90 ± 10.82 cm), showed notable enhancement in the moderately higher protein group. Muscle function and imaging assessments, such as handgrip strength and knee flexion, demonstrated improved functionality. MRI-derived measurements of the muscle composition of the calf (141.23 ± 4.87 MCSA, mm^2^ × 10^4^) and thigh (258.04 ± 7.26 MCSA, mm^2^ × 10^4^) further confirmed the positive impact of increased protein intake. The study concluded that a moderately high protein diet effectively supports muscle health in elderly females with sarcopenia. Therefore, an adequate protein intake may serve as a strategic nutritional intervention to mitigate muscle loss and improve overall physical function in aging women.

## 1 Introduction

Sarcopenia is a progressive condition characterized by the decline of skeletal muscle mass, strength, and physical function, primarily affecting older adults (1). This deterioration significantly reduces mobility, increases frailty, and elevates the risk of falls, fractures, and disability, ultimately leading to a lower quality of life and increased healthcare burden (2, 3). As people age, their body composition naturally changes, with fat mass increasing while lean mass decreases, leading to functional impairments (4). Since lean muscle mass is the body’s primary protein reservoir, it plays a crucial role in metabolic homeostasis, energy storage, and mobility (5). However, inadequate protein intake, along with hormonal imbalances, reduced physical activity, and chronic inflammation, accelerates muscle loss, worsening the progression of sarcopenia (6).

One of the most critical dietary factors influencing muscle preservation is protein intake. Adequate protein consumption supports muscle protein synthesis (MPS), strength maintenance, and recovery from muscle degradation (7). Essential amino acids (EAAs), particularly leucine, are pivotal for stimulating MPS, but older adults experience anabolic resistance, meaning they require higher protein intake to maintain muscle function (8). Current dietary guidelines recommend a daily protein intake of 0.8 g/kg body weight (RDA) (9), but emerging evidence suggests this is insufficient for preventing sarcopenia. Studies indicate that an intake of 1.0–1.2 g/kg body weight per day is more effective for preserving lean muscle mass, functional performance, and overall strength in older adults (10, 11).

A moderately high-protein diet (MHPD) has additional benefits beyond muscle preservation. It has been associated with improved weight management, reduced visceral fat accumulation, and lower waist circumference, which are particularly important for elderly individuals at risk of obesity-related metabolic disorders (12). High-protein diets also contribute to better glucose regulation, enhanced lipid metabolism, and increased satiety, making them a strategic nutritional intervention for healthy aging (13). However, despite these advantages, many elderly individuals, particularly women, fail to meet their daily protein requirements, increasing their vulnerability to muscle loss and physical decline (14).

Various diagnostic tools can assess muscle composition and function, allowing for early detection of sarcopenia. Magnetic resonance imaging (MRI) provides precise measurements of muscle cross-sectional area (MCSA), fat infiltration, and overall muscle quality (15). Handgrip strength tests serve as a reliable indicator of total muscle function, while knee flexion tests evaluate lower-limb strength and flexibility, both of which are critical for mobility and fall prevention (16).

This study aims to investigate the effect of different levels of dietary protein intake on muscle mass composition in elderly females with sarcopenia. It seeks to determine whether a moderately high-protein diet can serve as an effective nutritional strategy for mitigating sarcopenia and improving overall physical function in aging women.

## 2 Materials and methods

### 2.1 Ethics statement

The protocol was approved by the Research Ethical Committee of the University of Lahore (REC-UOL-583-11-2023). Participants submitted signed informed consent following a thorough description of the study’s goal, procedures, risks, and benefits. They were allowed to ask questions and informed that their participation was optional, with the ability to quit at any moment.

### 2.2 Study design

This research trial was conducted at the District Headquarters Hospital, Eid Gah Road, Okara, Pakistan. A total of 126 older females (60–75 years old) were enrolled and initially maintained their usual, unrestricted diets during baseline assessment. Participants were then randomly assigned to one of two dietary groups using a computer-generated randomization method in SPSS, with the allocation sequence concealed from the researchers responsible for recruitment and data collection to minimize selection bias. The groups included a normal-protein (NP) diet (15% of total energy from protein; *n* = 67) and a moderately higher-protein (MHP) diet (20–25% of total energy from protein; *n* = 67), both maintained for 12 weeks under an energy-restricted plan.

This study employed a single-blind design, where participants were unaware of their assigned dietary group to minimize bias in self-reported adherence and behavioral changes. However, due to the nature of dietary interventions, the researchers responsible for dietary monitoring and assessment were aware of the allocations. To control for potential confounding factors, baseline physical activity levels were assessed using the Physical Activity Questionnaire for the Elderly (PAQE), and participants were instructed to maintain their usual activity levels throughout the study. Additionally, metabolic parameters such as BMI, waist circumference, and body fat percentage were recorded at baseline to ensure comparability between groups. Participants with pre-existing metabolic disorders (e.g., diabetes, kidney disease) were excluded to minimize confounding effects.

Fat intake was kept constant at 25% in both diets, while carbohydrate intake was 60% for the NP group and 50% for the MHP group. The NP diet contained the recommended dietary allowance (RDA) of 0.8 g protein·kg^−1^·day^−1^, whereas the MHP diet provided 1.2 g protein·kg^−1^·day^−1^, corresponding to approximately 15 and 25% of total energy from protein, respectively, at the start of the intervention. Each participant’s diet was designed to provide 1,800 kcal/day, as detailed in Table 1.

**Table 1:** Macronutrient distribution and eating plan.

Factors	0.8 g/kg body weight/day protein group	1.2 g/kg body weight/day protein group
Macronutrient Distribution	Protein: ~15% of total energy intake Carbohydrates: ~60% Fats: ~25%	Protein: ~25% of total energy intake Carbohydrates: ~50% Fats: ~25%
Calories	1800 calories/day	1800 calories/day
Meal Plan (1 day plan) Breakfast	Oatmeal with milk, almonds, and honey	Scrambled eggs with whole-grain toast and avocado
Lunch	Grilled chicken with brown rice and steamed vegetables	Grilled salmon with quinoa and sautéed spinach
Dinner	Lentil soup with whole wheat bread and a side salad	Tofu stir-fry with mixed vegetables and brown rice
Food Preparation Methods & Protein Bioavailability	All meals were prepared using standardized methods to ensure consistency. Cooking techniques included boiling, steaming, grilling, and baking to preserve protein quality and minimize nutrient loss.	
Dietary Adherence Monitoring	24-h dietary recall on randomized days Regular follow-up sessions with a dietitian	

Elderly Sarcopenic female, age group (60–75 years), having handgrip strength test Asian Working Group for Sarcopenia (AWGS): (<18 kg) and knee flexion test (<0.90 Nm/kg), Muscle Cross-Sectional Area (MCSA) (Thigh MCSA: <40 cm^2^ and Calf MCSA: <25 cm^2^), (BMI > 25) were included. Females with kidney disease or autoimmune disorders that affect muscles or cause muscle degeneration (i.e., Myositis, Polymyositis) were excluded from the participation. Patients were monitored on different days using a 24-h dietary recall to confirm adherence to the interventional diet. At baseline and week 12th, physical tests were compared to assess the study hypothesis.

### 2.3 Methods of data collection

The sample size was estimated as a total of 126 participants (60–75 years) who met the study inclusion criteria and were enrolled and divided into two groups: normal protein intake (0.8 g/kg b.w/d) and moderately high protein intake (1.2 g/kg b.w/d) for 12 weeks (Table 2). By keeping a 20% dropout rate, we extended it to 134 (72 in each group).

**Table 2:** Treatment plan.

Plan	Control group T_1_	Treatment group T_2_
Diet type	Normal protein intake group (NP) 0.8 g/kg body weight	Moderately high protein diet (MHP) 1.2 g/kg body weight
Participants	63 (20% Expected dropout rate)	63 (20% Expected dropout rate)
Duration	12 weeks	
Target group	60–75 years	

#### 2.3.1 Anthropometric measurement

The baseline data for anthropometric measures, such as weight, height, BMI, waist circumference, and fat mass, were obtained using the protocols described in the previous study (17). Weight was measured using a calibrated digital scale, with subjects wearing light clothes and no shoes. Participants’ heights were measured with a stadiometer while standing erect, facing straight ahead, and wearing no shoes. BMI was computed by dividing weight (kg) by height squared (m^2^). Waist circumference was measured using a flexible tape measure at the halfway between the lower rib and the iliac crest, after a typical exhale. A bioelectrical impedance analysis (BIA) instrument was used to determine fat mass (17).

#### 2.3.2 Muscle function and composition assessment tests

Handgrip strength, knee flexion/extension tests, and Magnetic Resonance Imaging (MRI) of the thigh and calf were used to measure muscle function and composition, as previously reported (11). Handgrip strength was assessed using a Jamar hydraulic hand dynamometer, with participants completing three maximum squeezes with their dominant hand and recording the greatest value. The knee flexion/extension test was performed using a dynamometer, with individuals completing both knee flexion and extension movements against resistance, and the maximal force generated during each test was recorded. MRI of the thigh and calf was done using a 3 T MRI scanner, with subjects lying supine and their legs at a standard angle to guarantee consistency. Cross-sectional pictures of the thigh and calf muscles were taken, and muscle composition, including muscle mass and fat infiltration, was calculated using specialized software (11, 17).

### 2.4 Statistical analysis

All data were analyzed utilizing the most recent version of SPSS 25. Descriptive statistics (mean ± standard deviation) were used for continuous variables, and frequencies and percentages were used for categorical variables. To compare continuous variables between the two groups, an independent t-test was used for age, as it was treated as a continuous variable. Kolmogorov–Smirnov Test was used to check the normality of the data Baseline and post-study findings were analyzed using a paired sample t-test with a significance level of *p*-value <0.05 (18).

## 3 Results

### 3.1 Demographic and socioeconomic characteristics

The study included 126 elderly females (60–75 years old) with sarcopenia, who were randomly assigned to two groups: the normal protein intake group (0.8 g/kg b.w/day; *n* = 63) and the moderately high protein intake group (1.2 g/kg b.w/day; *n* = 63). Both groups had a similar mean age (68.8–69.5 years) and were 100% postmenopausal. Marital status was comparable, with the majority being married (65% in the normal protein group vs. 68% in the high-protein group).

Regarding socioeconomic factors, 50% of participants in both groups belonged to the middle class, while the high-protein group had a slightly higher proportion of individuals with secondary education or above (75% vs. 70%). Although employment status was similar between groups (approximately 5% employed), knowledge of sarcopenia and protein intake differed significantly (*p* = 0.038 and *p* = 0.028, respectively), with higher awareness in the high-protein group. Comorbidities were present in 79–83% of participants, but there was no significant difference between groups (*p* = 0.684). Similarly, there was no significant difference in the use of bone-related medications (*p* = 0.569) (Table 3).

**Table 3:** Demographic and clinical characteristics of the participants.

Characteristics	0.8 g/kg body weight/day protein group T1	1.2 g/kg body weight/day protein group T2	*p*-value
Age (years)	68.8 ± 4.61	69.5 ± 2.30	0.482
Marital Status (%)	Married: 65% Widow: 35%	Married: 68% Widow: 32%	0.631
Menopause Status (%)	Postmenopausal: 100%	Postmenopausal: 100%	
Socioeconomic Status (%)	Low: 30% Middle:50% High:20%	Low: 35% Middle:50% High:20%	0.521
Education level (%)	Primary:30% Secondary & above:70%	Primary:25% Secondary & above:75%	0.317
Employment Status (%)	Employed:5% Umemployed:95%	Employed:4% Umemployed:96%	0.432
Comorbidities (%)	Yes: 83% No: 17%	Yes: 79% No: 21%	0.684
Knowledge about protein intake (%)	Yes:30% No: 70%	Yes:35% No: 65%	0.028*
Knowledge about Sarcopenia (%)	Aware: 25% Unaware: 75%	Aware: 30% Unaware: 70%	0.021*
Taking Bone-related Medication (%)	Yes: 45% No: 55%	Yes: 48% No: 52%	0.569

### 3.2 Effect of protein intake on waist circumference

After 12 weeks, participants in the moderately high-protein diet (MHPD) group exhibited a significant reduction in waist circumference compared to those in the normal protein intake (NP) group. The mean waist circumference decreased from 117.80 ± 11.21 cm to 113.90 ± 10.82 cm in the MHPD group (*p* = 0.001), while the NP group showed a smaller reduction from 116.83 ± 8.31 cm to 114.53 ± 8.05 cm (*p* = 0.006) (Table 4).

**Table 4:** Effect of protein diet intake on waist circumference (cm) among elderly females.

Waist circumference		Mean ± S.D	Std. Error Mean	*p*-value
Normal protein intake group T1	Initial	116.83 ± 8.31	1.532	0.006
	Final	114.53 ± 8.05	1.486	
Moderately High Protein Diet T2	Initial	117.80 ± 11.21	1.991	0.001*
	Final	113.90 ± 10.82	1.975	

*Indicates statistical significance as determined by paired sample t-tests.

### 3.3 Effect of protein diet intake on body fat (kg) among elderly females

A significant reduction in fat mass (kg) was observed in both groups, but the reduction was greater in the high-protein group. The MHPD group experienced a fat mass reduction of 2.96 kg, from 33.67 ± 4.91 kg to 30.71 ± 5.16 kg (*p* < 0.001). In contrast, the NP group had a reduction of 1.28 kg, from 33.69 ± 5.46 kg to 32.41 ± 5.44 kg (*p* = 0.046) (Table 5).

**Table 5:** Effect of protein diet intake on body fat (kg) among elderly females.

Body fat		Mean ± S.D	Std. Error Mean	*p*-value
Normal protein intake group T1	Initial	33.69 ± 5.46	0.997	0.046*
	Final	32.41 ± 5.44	0.994	
Moderately High Protein Diet T2	Initial	33.67 ± 4.91	0.897	<0.001*
	Final	30.71 ± 5.16	0.942	

*Indicates statistical significance as determined by paired sample t-tests.

### 3.4 Effect of protein diet intake on knee flexion/extension (Nm/kg) among elderly females

The knee flexion strength (Nm/kg) improved significantly after 12 weeks in both groups. Participants in the MHPD group had a greater increase, from 0.52 ± 0.18 Nm/kg to 0.93 ± 0.50 Nm/kg (*p* < 0.001), while the NP group showed an improvement from 0.50 ± 0.14 Nm/kg to 0.72 ± 0.13 Nm/kg (*p* = 0.006) (Table 6).

**Table 6:** Effect of protein diet intake on knee flexion/extension (Nm/kg) among elderly females.

Knee flexion		Mean ± S.D	Std. Error Mean	*p*-value
Normal protein intake group T1	Initial	0.50 ± 0.14	0.017	0.006*
	Final	0.72 ± 0.13	0.016	
Moderately High Protein Diet T2	Initial	0.52 ± 0.18	0.020	<0.001*
	Final	0.93 ± 0.50	0.063	

*Indicates statistical significance as determined by paired sample t-tests.

### 3.5 Effect of protein diet intake on handgrip (kg) among elderly females

Handgrip strength (kg) significantly improved in both groups. In the MHPD group, grip strength increased from 18.12 ± 1.04 kg to 21.46 ± 1.06 kg (*p* < 0.001). The NP group also showed improvement, from 18.19 ± 1.85 kg to 20.52 ± 3.83 kg (*p* = 0.004) (Table 7).

**Table 7:** Effect of protein diet intake on handgrip (kg) among elderly females.

Handgrip		Mean ± S.D	Std. Error Mean	*p*-value
Normal protein intake group T1	Initial	18.19 ± 1.85	0.231	0.004*
	Final	20.52 ± 3.83	0.482	
Moderately High Protein Diet T2	Initial	18.12 ± 1.04	0.131	<0.001*
	Final	21.46 ± 1.06	0.133	

*Indicates statistical significance as determined by paired sample t-tests.

### 3.6 Effect of protein intake on MRI- derived measurements of muscle composition

**Figure 1:**
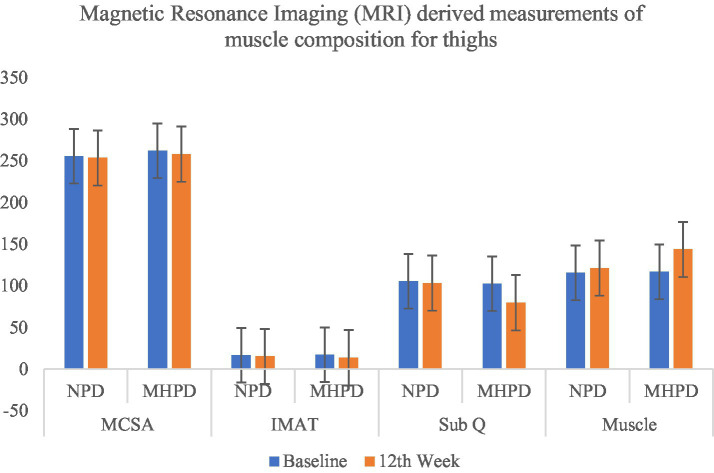
Effect of moderately high protein intake on Magnetic Resonance Imaging (MRI) derived measurements of muscle composition for thighs among groups. NPD, Normal Protein Diet; MHPD, Moderately High Protein Diet; MCSA, Muscle Cross Sectional Area; IMAT, Intermuscular Adipose Tissue; Sub Q, Subcutaneous Fat.

**Figure 2:**
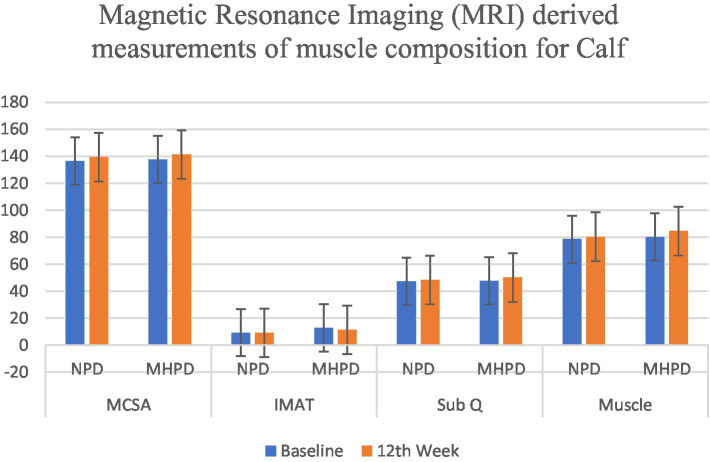
Effect of moderately high protein intake on Magnetic Resonance Imaging (MRI) derived measurements of muscle composition for Calf among groups. NPD, Normal Protein Diet; MHPD, Moderately High Protein Diet; MCSA, Muscle Cross Sectional Area; IMAT, Intermuscular Adipose Tissue; Sub Q, Subcutaneous Fat.

MRI analysis revealed significant improvements in muscle mass composition in the MHPD group compared to the NP group. The calf muscle cross-sectional area (MCSA) increased to 141.23 ± 4.87 mm^2^ × 10^4^, while the thigh MCSA improved to 258.04 ± 7.26 mm^2^ × 10^4^ in the MHPD group. Furthermore, intermuscular adipose tissue (IMAT) and subcutaneous fat (SubQ) decreased in both muscle groups, indicating improved muscle quality (Figures 1, 2).

## 4 Discussion

This study investigated the effects of different levels of protein intake on muscle mass composition, strength and overall body composition in elderly females with sarcopenia. The findings indicate that a moderately high-protein diet (1.2 g/kg body weight/day) led to significant improvements in muscle strength, reduced fat accumulation, and enhanced muscle composition compared to the normal-protein intake group (0.8 g/kg body weight/day). These results reinforce the growing evidence that higher protein intake is crucial for maintaining muscle function and preventing sarcopenia-related muscle deterioration (10, 11). Protein is essential for muscle protein synthesis, particularly in older adults who experience anabolic resistance, necessitating higher protein intake to maintain muscle integrity (8). The observed improvements in muscle mass and strength in the high-protein group align with findings from previous studies that suggest increasing dietary protein beyond the recommended daily allowance (RDA) can help counteract age-related muscle loss (7, 28).

One of the key findings of this study was the significant reduction in waist circumference and fat mass in the moderately high-protein diet group. Waist circumference is a critical indicator of metabolic health and its reduction suggests an improvement in body composition. The greater decrease in waist circumference (−3.9 cm) and fat mass (−2.96 kg) in the high-protein group compared to the normal-protein group (−2.3 cm and −1.28 kg, respectively) suggests that protein intake plays a key role in fat metabolism and energy balance (12). Prior research has demonstrated that higher protein intake enhances satiety, increases energy expenditure and promotes fat oxidation, which can lead to reductions in visceral fat accumulation (13, 19). The observed improvements may also be attributed to protein-induced thermogenesis, improved insulin sensitivity and enhanced lipid metabolism, all of which contribute to better body composition and reduced adiposity in aging populations (9, 29).

The significant improvements in muscle strength, as assessed by knee flexion and handgrip tests, further highlight the benefits of a higher protein intake. The increase in handgrip strength (21.46 ± 1.06 kg) and knee flexion strength (0.93 ± 0.50 Nm/kg) in the high-protein group was greater than the improvements observed in the normal-protein group. These results are consistent with previous findings that dietary protein stimulates the mechanistic target of rapamycin (mTOR) signaling pathway, which regulates muscle protein synthesis and prevents muscle loss (20, 30, 31). Aging is associated with reduced responsiveness to anabolic stimuli, making dietary protein intake even more critical for muscle maintenance (8, 26, 27). The improvements in strength observed in this study align with research showing that protein supplementation combined with resistance training enhances muscle function and physical performance in older adults (17, 21).

Magnetic resonance imaging (MRI) provided further evidence of muscle quality improvements in the high-protein group. The increase in muscle cross-sectional area (MCSA), along with the reduction in intermuscular adipose tissue (IMAT) and subcutaneous fat, suggests that higher protein intake enhances muscle composition while reducing fat infiltration (22). The reduction in IMAT is particularly noteworthy, as excess fat accumulation within muscle tissue is linked to reduced muscle strength, insulin resistance, and poor functional outcomes in older adults (23, 32). Previous studies have shown that higher protein intake is associated with better muscle morphology, including increased lean mass and lower fat infiltration, which supports the findings of this study (24, 25, 33).

This study has several notable strengths. First, it employs a randomized study design, minimizing selection bias and ensuring balanced baseline characteristics between groups. Second, objective assessments such as MRI-derived muscle composition analysis, handgrip strength and knee flexion tests provide reliable measures of muscle function and composition, reducing measurement bias. Third, dietary intake was controlled through standardized meal plans, ensuring consistency in protein consumption across participants. Additionally, the use of a single-blind design, where participants were unaware of their group allocation, helps minimize bias in adherence and self-reported outcomes. These factors contribute to the robustness of our findings.

## 5 Limitations and recommendations

1. **Absence of a Placebo Group and binding constraints**: This study did not include a placebo group, which could have further strengthened causal inferences. Additionally, due to the nature of dietary interventions, double-blinding was not feasible and participants were aware of their assigned diets. However, to minimize performance bias, objective, validated assessment methods (e.g., MRI-derived muscle composition analysis, handgrip strength tests, and knee flexion tests) were employed, and dietary intake was strictly controlled to ensure comparability between groups. Future research should explore alternative methodological approaches, such as crossover designs or more extended intervention periods, to further establish causality.
2. **Short Study Duration**: The 12-week intervention period may not have been long enough to observe the long-term effects of protein consumption on muscle mass, strength, and sarcopenia progression. Longer follow-up durations are needed to assess the sustained benefits of increased protein intake and its potential role in preventing sarcopenia-related complications over time.
3. **Self-reported Dietary Intake and Compliance Monitoring**: Dietary adherence was assessed using 24-h dietary recalls, which are prone to recall bias and underreporting. To improve dietary compliance evaluation, future studies should consider multiple 24-h recalls on non-consecutive days, incorporating food diaries for cross-validation. Additionally, implementing participant training in portion estimation, dietitian-supervised recalls, and digital meal tracking applications may enhance data reliability and reduce reporting errors.
4. **Population and Geographic Limitations**: This study was conducted within a specific population and geographic region, which may limit the generalizability of the findings to other ethnic, socioeconomic, and lifestyle groups. Future research should consider multi-center trials with larger and more diverse populations to enhance external validity and applicability across different demographic settings.

## 6 Conclusion

This study provides strong evidence that a moderately high-protein diet (1.2 g/kg body weight/day) is more effective than the standard recommended intake (0.8 g/kg body weight/day) in preserving muscle mass, enhancing strength, and improving body composition in elderly females with sarcopenia. The findings demonstrated significant reductions in waist circumference and fat mass, coupled with notable gains in muscle strength and cross-sectional muscle area, particularly in the thigh and calf muscles. The MRI-derived improvements in muscle composition, including reduced intermuscular adipose tissue and subcutaneous fat, suggest that increasing protein intake not only prevents muscle loss but also enhances muscle quality and metabolic health.

Given the growing burden of sarcopenia and age-related muscle decline, these results highlight the need for revising dietary protein recommendations for elderly individuals. Current guidelines may not be sufficient to counteract anabolic resistance and muscle degradation in aging populations. A protein intake of at least 1.2 g/kg body weight/day should be considered as part of a targeted nutritional intervention for preventing muscle deterioration, maintaining functional independence, and reducing the risk of falls and frailty in older adults.

Furthermore, the study emphasizes the importance of comprehensive dietary strategies that incorporate both animal- and plant-based protein sources to ensure a balanced intake of essential amino acids. Future research should explore long-term interventions combining protein intake with resistance training and other lifestyle modifications to optimize muscle preservation and functional performance in aging populations.

In light of these findings, public health policies should promote adequate protein consumption as a key strategy for healthy aging. Increased awareness, personalized dietary guidance and community-based interventions can play a crucial role in mitigating the adverse effects of sarcopenia, ultimately improving the quality of life and overall well-being of elderly individuals.

## Data Availability

The raw data supporting the conclusions of this article will be made available by the authors, without undue reservation.
